# Two sides of the story: bridging organizational and individual resilience - a qualitative study

**DOI:** 10.1186/s12913-025-13013-z

**Published:** 2025-08-09

**Authors:** Hilda Bø Lyng, Colleen Cheek, Cecilie Haraldseid-Driftland

**Affiliations:** 1 SHARE – Centre for Resilience in Healthcare, Faculty of Health Sciences, University of Stavanger, Stavanger, Norway; 2 Australian Institute of Health Innovation, Macquarie University, Sydney, Australia

**Keywords:** Autonomy, Competence, Relatedness, Individual resilience, Organisational resilience, Champions, Adaptation

## Abstract

**Background:**

In resilience literature, champions are recognized as key organizational resources. Yet, their willingness to take on these roles and contribute extra efforts is rooted in autonomous motivation. This study aims to contribute understanding of both sides of this resilience story through the exploring of the motivation of champions found to contribute to organizational resilience in healthcare.

**Methods:**

The empirical setting for this study includes eight different sites (two hospitals, five nursing homes and one home healthcare service) entailing interviews and observations of 55 healthcare informants (leaders and workers). Analysis of collected qualitative interviews through a deductive -inductive thematic analysis, using Self-determination Theory as an a priori framework for the deductive analysis.

**Results:**

The importance of fostering connection among team members as well as between team members and leaders, offering flexibility to the champion to manage their role, and brokering relationships among teams or departments were illuminated.

**Conclusions:**

Through the exploration of champions’ autonomous motivation for acting as organizational champions to ensure resilient performance in healthcare organizations, this study provides a more holistic picture where both sides of the story can be understood. Such holistic knowledge of champions is key to understand how to identify, support and maintain champions in their role to lay the groundwork for resilient performance in healthcare.

**Supplementary Information:**

The online version contains supplementary material available at 10.1186/s12913-025-13013-z.

## Background

Focus on strengthening resilience of healthcare organizations has increased over the last decades [1]. Global health emersychological needs theorygencies like the recent COVID-19 pandemic have catalysed the urgency of strengthening resilience in healthcare even more in order to prepare, handle and overcome challenges in healthcare organisation and delivery [2]. Resilience in healthcare is understood as *“the capacity to adapt to challenges and changes at different systems levels to maintain high quality care”* ([1], p.6).

Champions have been found to act as a key organization capacity for ensuring resilience in healthcare [3]. Organizational champions are individuals who display commitment, passion and engagement for a specific cause or initiative and are important as an organizational resource through their actions [4, 5]. Even though they may not hold a formal leadership role, champions lead in informal ways, applying their expertise and motivating others toward achievement of organizational goals [3, 6, 7]. As champions often emerge in informal ways, the organization may or may not identify this resource, so they are not necessarily intentionally or evenly distributed throughout the organization [3].

For champions to thrive in the organization and perform their magic, champions need to be identified, acknowledged, and provided ‘room for manoeuvre’. In this context, ‘room for manoeuvre’ derives from social theories and refers to the extent of choice that is transferred from a central level to a local level, in bureaucratic organisations [8]; such empowerment is an important enabler of champions assuring resilient performance [3, 9, 10]. At a local level, champions advocate for an initiative and move beyond their usual work roles to engage in planning and implementation, and importantly, motivating others, to accomplish or adapt to change [4, 5].

While champions significantly contribute to organizational resilience, they are individuals who require motivation to take on this role and exert the extra effort needed [11]. Support and engagement from leaders are crucial for champions to sustain their energy and proactive initiatives. However, such support is not always available or enough. At this time champions must rely on their own motivation to act. Leaders need understanding of how to support autonomous motivation of champions to sustain resilient performance [11].

While champions are recognized as an organizational resource in the resilience literature [3], there are two sides to this story. For champions to willingly take on their roles and contribute extra efforts to the organization, they must be autonomously motivated [11]. This study aims to explore the autonomous motivation of champions who contribute to ensuring organizational resilience in healthcare. The overarching goal is to provide more holistic understanding of the role of champions in strengthening resilient performance in healthcare settings. These links between individual actors within the system and organizational resilience are unexplored and understudied, despite clear evidence that they influence and complement each other [12].

## Theoretical framework

Self-determination theory (SDT) is a valuable theoretical framework for understanding and describing both individual and organizational consequences, making it particularly useful for exploring the aim of this study. SDT is a socio-cognitive theory, derived from motivation research [13]. SDT posits the impact of organisational factors (such as job design, managerial styles, pay) on workers motivations and experiences is mediated by three basic psychological need constructs: the need for autonomy, for competence, and for relatedness (Table 1) [14]. SDT posits that fulfilment of all three are essential for individual employee adjustment, wellbeing, and psychological growth [15], promoting autonomous motivation and high-quality performance [16]. There is considerable literature that demonstrates autonomous motivations (characterised by individuals being engaged in an activity with a full sense of willingness, volition and choice) optimises employee functioning (performance, well-being, workplace civility) more than controlled motivations (characterised by individuals’ reluctant engagement, often through compulsion) [16, 17]. Further, it has been shown that even when the organization is not supportive, clinicians draw from autonomous motivation to provide good patient care as a mediator for ongoing clinical performance [11].

**Table 1:** Basic psychological needs according to Basic Psychological Needs (Self Determination Theory [14]

Autonomy refers to the experience of volition and authenticity in an individual’s thoughts, feelings, and actions. Need frustration refers to the experience of inner conflict and pressure.
Competence refers to the experience of effectiveness and mastery. Need frustration refers to the experience of a sense of failure and helplessness.
Relatedness refers to the experience of warmth and connectedness to others. Need frustration refers to the experience of social alienation and exclusion.

### Design and setting

The study made use of an explorative qualitative design, including both-semi structured focus group interviews pre- and post-intervention and observations from workshops in the data collection process.

**Fig. 1:**
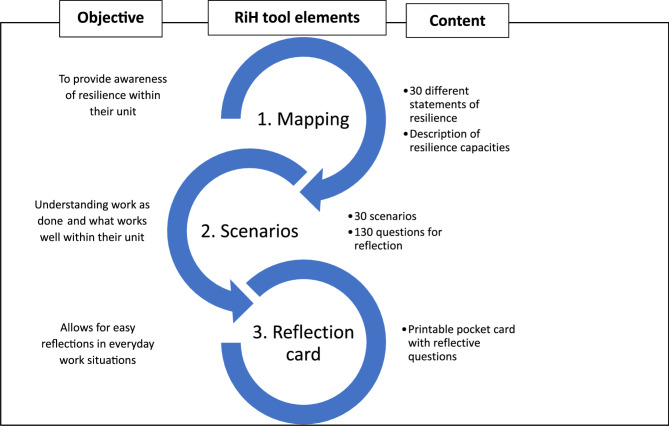
Description of the RiH learning tool [18]

This study is a part of the Resilience in Healthcare (RiH) project, opting to strengthen the Norwegian healthcare system through the development and implementation of a resilience learning tool to translate resilience to clinical practice. The resilience learning tool used in this study is the Resilience in Healthcare Reflection tool. The tool is comprised of three different elements. Element 1: ‘Mapping’, which aims to provide awareness of resilience within the participating unit; Element 2: ‘Scenarios’, which aims to create understanding among the participants of what works well and why in their unit; and Element 3 ‘Reflection list’ which aims to allow for short reflections over own practice in everyday work situations. Please see overview in Fig. 1. The full version of the tool can be found in both Norwegian and English at: https://rih.uis.no/. For information about the development process of the tool, please see [18, 19].

The RiH learning tool was implemented and evaluated in nine different healthcare sites across 27 different units over four months. The intervention design was based on a train-the-trainer methodology [20] where 1 or 2 representatives from each unit was trained by researchers in how to use the different sections of the tool (55 representatives in total). The TTT staff then participated in different learning tool related assignments with their colleagues at each respective unit. For TTT workshop no.1, the TTT staff was trained in how to use the ‘mapping’ section of the tool; for TTT workshop no.2, the TTT staff was trained in how to use the ‘scenarios’ section in the tool; and for TTT workshop no.3, the TTT staff was trained in how to use the ‘reflection list’ in the tool. Between each TTT workshop, the TTT staff was asked to undertake two different assignments. Both assignments corresponded with the respective topic for the previous TTT workshop. Focus group interviews were held pre and post implementation, while observations was done during each TTT Workshop, as depicted in Fig. 1. No data collection was gathered during the different task assignments.

**Fig. 2:**
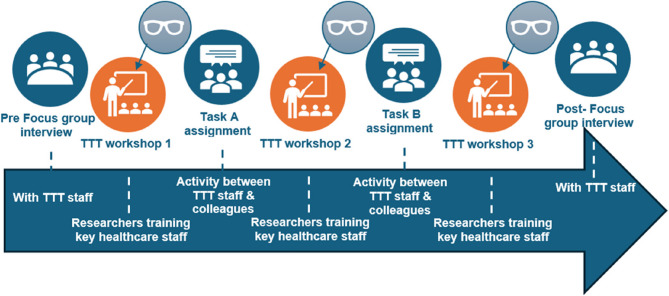
Overview of the intervention design and activities for all participating units

The nine different Norwegian healthcare sites, comprised six nursing homes, two hospitals and one homecare service, tested the RiH learning tool. The 27 different units were located in five different municipalities of varying sizes, ranging from rural municipalities to big cities. Five nursing homes were owned by a private not-for-profit enterprise in a large city; the sixth was a public nursing home in a small municipality. Both the hospitals and the home care service were publicly funded. The inclusion of different healthcare settings and a diversity of locations allowed for more holistic understanding than seeking to explore a specific, homogenous setting.

### Participants

The senior management from each of the units accepted to participate in the implementation decided themselves who were going to be the staff representing their unit in the intervention and as such become the TTT staff that were responsible for participating in all workshops and carry out the designated activities. A total of 55 participants were chosen, 1–3 staff from each unit to ensure representation at all workshops and activities. There were no requirements to who could become the unites representative, some units therefore chose the senior managers themselves, while others selected other types of leaders such as management assistants or team leaders, or registered nurses or assistants. All 55 were invited to contribute to all of the data collection activities and attended based on their availability, with a minimum of one representative from each unit at all activities.

### Data collection

#### Pre- and post-focus group interviews

Eight Semi-structured focus group interviews were performed pre -intervention (*n* = 47) and six focus group interviews were held post-intervention (*n* = 32), see Fig. 2, and supplementary file 1 and 2. The difference in number of informants between the pre- and post-focus group interviews was due to workload at their respective units. The data collection took place in later stages of the COVID − 19 pandemic, and as many of the included units were at times heavily impacted staff were unable to leave their work to take part in the focus group interviews. All focus group interviews lasted for 60–90 min and at least 2 researchers were present in all focus group interviews. Focus group interviews were audio recorded and transcribed verbatim. The subject of the pre-intervention focus group interviews was the description of current work practices according to the ten different capacities for resilience (structure, competence, learning, alignment, coordination, risk awareness, leadership, involvement, champions, and communication) [3]. Topic of post intervention focus group interviews included participant experiences of using the learning tool and the implementation process.

#### Observations

30 h of observations were performed at a total of 15 workshops, one for each of the TTT workshops that were held. All participating units attended a set of three different TTT workshops, time and place for their attendance were based upon geographical location and availability (see Fig. 2 for overview of all activities). In total workshop one had 39 participants, workshop two had 31 participants and workshop three had 32 participants). Between 2 and 4 researchers took part in the observation of all workshops, and observation notes were collected through a template to ensure consistency across the sites. Observations were used as means to identify particulars about the work taking place [21] as well as interactions and dynamics when using the tool and between the participants and with the tool (see supplementary file 3). Thirty hours of observations of the 15 workshops were included and described in a pre-design observation template.

All data collection was conducted in Norwegian.

### Data analysis

In this sub-analysis all translated focus group interviews were reanalysed using the six-step routine of a deductive-inductive thematic analysis by Braun and Clarke [22].

All transcribed focus group interviews and observation notes were included in the analysis. As the focus of this paper is to better understand the motivation of champions in terms of contributing to organizational resilience, questions directly concerning the role of champions made up most of the dataset for this study. Champions is one of the resilient capacities [3] addressed in the interview guide using the questions: Are there individuals in your organization who take on extra responsibilities and tasks? Are there individuals in your organization that engage in training of others? How are champions identified in your organization? How does the unit support these champions in their work? What is the role of champions in your unit? All data were included as the informants spoke about champions during their responses to other questions.

The data analysis followed the six steps of thematic analysis. Step one ‘*Familiarize yourself with the data’*: Both author CHD and HBL re-read all transcripts, noting down and discussing initial ideas with author CC. It became evident that the data were suitable to be reanalysed deductively according to the SDT constructs of autonomy, competence, and relatedness. An additional code for data related to motivation but not in accordance with the three concepts of SDT was included.

In the second round of analysis, conducted in accordance with the SDT framework, the dataset was explored from a different perspective. Champions are found important for resilient performance and as an organizational resource in the resilience in healthcare literature [3]. By examining the motivation of champions to act as an organizational capacity, we gained insights into how to support these champions effectively.

Author HBL led steps two and three ‘*Generating initial code’s* and ‘*Searching for themes’*: Participant perceptions were coded deductively to one or more of the three constructs, autonomy, competence, and relatedness, and the ‘other’ code. When the first deductive round of analysis was completed, a second round of analysis was completed whereby the data in each of the codes/themes were inductively analysed to explore what these themes entailed for this study. All inductive themes were then organised into categories within each SDT construct. Emerging inductive themes under the relatedness concept included: *‘Engagement culture’*, *‘Aligning organizational and individual goals’*, and *‘Relationship broker’. Inductive themes for the autonomy concept included: ‘Flexibility’* and *‘Initiating’*,* Inductive themes for the Competence concept included ‘Knowledge sharing’ and ‘Experts as resources’* and the other category included the inductive theme ‘*Personal characteristics’.*

Step four *Reviewing themes*: Authors CHD and HBL separately reviewed the inductive themes to make sure the work stood in relation to the coded extracts.

Steps five and six *Defining and naming the themes* and *producing the repor*t: all authors collaborated to define themes further and author CC assisted with interpretation and semantic translation to English to preserve, not change, initial meaning.

## Results

**Fig. 3:**
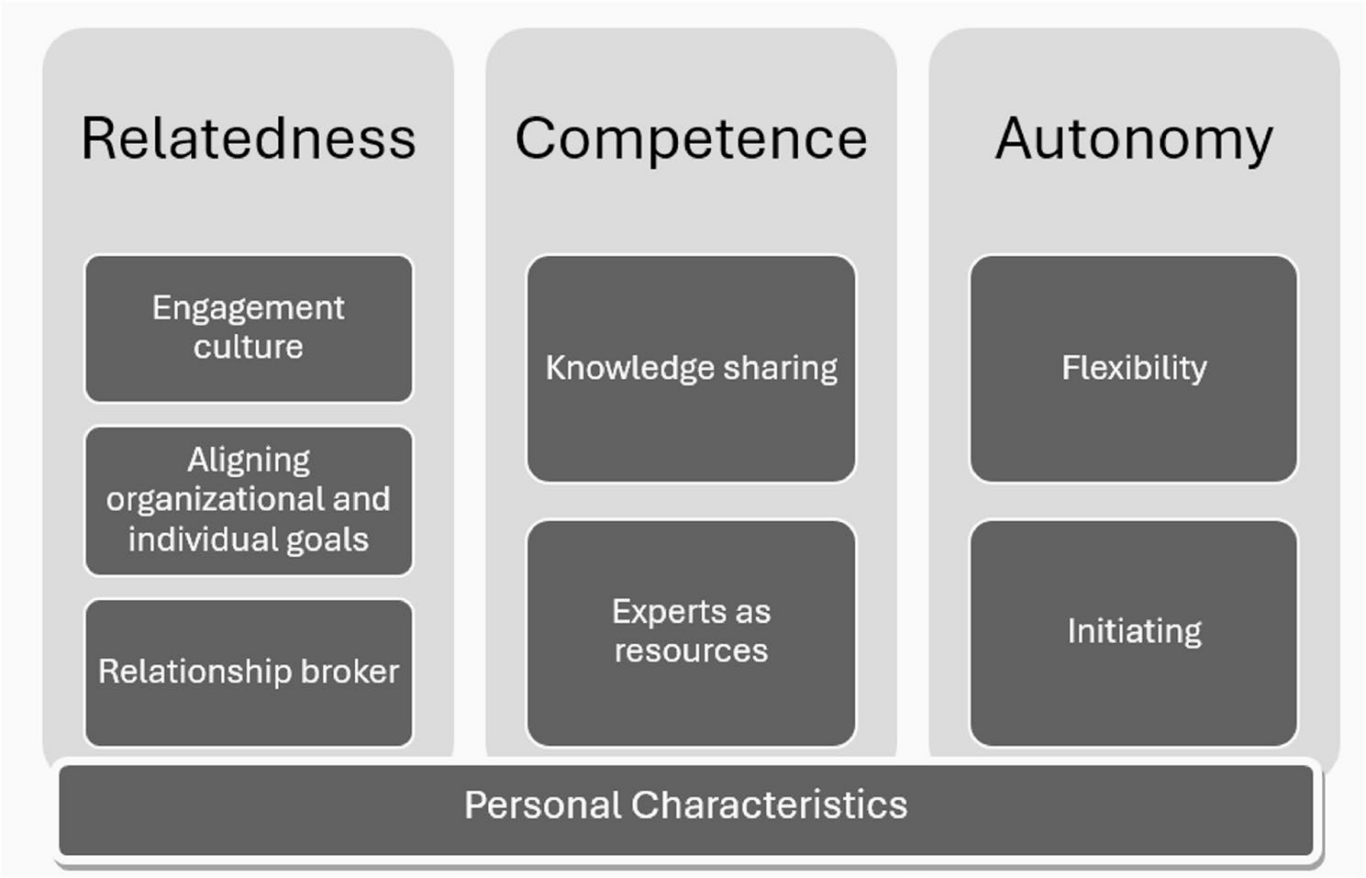
Overview of study findings

We deductively mapped the pre- and post-intervention data on champions to the three constructs Relatedness, Autonomy and Competency. All concepts of the SDT framework were reflected in all of the focus-group interviews. The concept of relatedness was the most frequently observed, with 71 instances in the dataset. Competence was noted 34 times, autonomy 12 times, and personal characteristics 26 times. In addition, we found reference to the personal characteristics of champions in the inductive analysis. These are summarised below, illustrated with representative quotes, Fig. 3.

### Relatedness

Three main sub-themes of relatedness, categorised as *‘Engagement culture’*, *‘Aligning organizational and individual goals’*, and *‘Relationship broker’* were identified.

#### Engagement culture

Champions were described as essential for the development of a positive work culture. Champions acknowledged their colleagues warmly and spoke productively of their abilities and personal characteristics to others. Positive work culture fostered colleague’s and team member’s confidence to share more informally and openly about themselves and their interests, strengthening relationships within the team.

*“So*,* I try to pass on nice things I hear about the others. Because we often say nice things about each other….like* [name] *is so good at baking. So*,* I tell her: “I think you are great at baking”. Having this kind of culture is important”.* (Focus group interview, Nursing home, Pre intervention, Site 4).

The development of good working relationships among the team was considered to be important for champions to thrive in their work and their role. To strengthen these relationships, leaders strived to be present at the frontline to engage with champions and the other team members. Leaders also monitored champions well-being and actively encouraged them to sustain their motivation. While there were no explicit expressions of need frustration in the data, perceptions such as the one below illustrate how need frustration may manifest without the required leader support.

*“To be able to balance it* [champion’s motivation], *and to support the champions. Because we have many champions. But it does not take many unfortunate situations before the “flame” is strangled. So*,* to nurture the champions is a leader responsibility.”* (Focus group interview, Hospital, Pre-intervention, Site 3).

#### Aligning organizational and individual goals

Champions, either acting formally in an assigned role (like a coordinator role) or informally in a tacit role, needed to be recognized and appreciated for their extra initiatives. Champions were easily engaged in higher-level discussions, decisions, and tasks as they were highly motivated and often highly knowledgeable. As such they were a discussion partner for their leaders. Since the needs of the organization and the needs of the champions did not always align, the leader must guide champions in the direction they perceived to be most correct. For example, in a situation where a champion requested time to explore and learn more about a subject that was not perceived to provide direct value for the organization, the leader denied this request:

*“What I feel is sensitive*,* is when someone perceives themselves to be a champion and shows a huge interest in something. But the department and the colleagues will not get much out of it.”* (Focus group interview, Hospital, Pre intervention, Site 3).

#### Relationship broker

Champions were often found to assume the role of a broker or negotiator between different departments, between the hospital and a nursing home, or between the healthcare institution and the patient’s home. Actively cultivating cooperation and information transfer between healthcare professionals, patients, next-of-kin and other stakeholders, assisted seamless service integration among all partners involved in the patients’ overall care:

*“I would say that the main idea with this role is that the person should act as a middleman between the department*,* the patient’s home*,* and the hospital”* (Focus group interview, Nursing home, Pre intervention, Site 2).

### Competence

Two sub-themes were categorised of Competence, categorised as *‘Knowledge sharing’* and *‘Experts as resources’* were identified.

#### Knowledge sharing

Informants recognized that having highly knowledgeable and competent people did not convey benefit for the organization unless their knowledge was shared with others. As such, champions were key stakeholders who actively engaged in knowledge sharing, education and training of students and new colleagues, and thus were found highly valuable for increasing the competence of the organization.

*“I would like to emphasize the person who later on always sends an article on the topic we discussed*,* without being asked to do so. She never says it out loud*,* but she sends the email to all of us*,* because she found an article on exactly what we discussed during lunch”* (Focus group interview, Nursing home, Post intervention Site 4 bcd).

#### Experts as resources

Leaders and colleagues within the organization actively directed colleagues in need of assistance towards the “expert/champion” of the group. The rewards of this practice were threefold: the knowledge of who to contact for clinical support in the organization was strengthened and more widely disseminated; recognition of the champion as an expert motivated champions to maintain or expand on their current knowledge, improving the quality of knowledge transfer to less competent peers; and motivated champions modelled enthusiasm and interest, fuelling intrinsic motivation in others.

*“To give them opportunities and to support them. Then they get very inspired and want to develop themselves further. And this* [motivation] *is “infectious” for others as well”* (Focus group interview, Hospital, Pre intervention, Site 3).

### Autonomy

Two sub-themes of Autonomy categorised as *‘Flexibility’* and *‘Initiating’* were identified.

#### Flexibility

Champions expressed the need for flexibility in the way they prioritised and handled their work tasks, to maintain their engagement and motivation. Leaders sometimes found it difficult to provide champions with this flexibility, due to limited time and resources available in the organization.

*“There is little room for flexibility at our place. Most things are going into the day-to-day work. There are incoming referrals*,* and you need to deal with them. Some patients are to be discharged*,* and some patients are deteriorating. It is the totality of all these things”* (Focus group interview, home care service, Pre intervention, Site 5).

However, it was emphasized that without flexibility champions were found prone to burnout. Some champions managed to increase their autonomy by taking on additional activities by choice, such as courses in their spare time. Others possessed a detailed knowledge of the system, which allowed them to create spaces for flexibility:

*“It is always best when the champion has their own defined area. And if they also understand the system a bit*,* so that they understand how and where they can use their “champion energy” it is even better”* (Focus group interview, Hospital, Pre intervention, Site 3).

#### Initiating

Champions provided support to their peers through a range of different initiatives. Some champions contributed to the social environment and initiated birthday parties and other forms of social arrangements (e.g., National holidays). Others willingly contributed their knowledge and expertise when others needed help. Appreciating this variation among the champions was considered an important task for leaders and colleagues, to nurture and retain champions:

*“They are great in following up on birthdays in my department. Both for the patients and for colleagues. My impression is that there is almost constantly a fundraising for birthday presents. So that all who work at the department get a little gift on their birthday. And there is always singing. There are a just few employees who facilitate all of this”* (Focus group interview, Nursing home, Pre intervention, Site 2).

### Other

#### Personal characteristics

Champions were described as individuals who saw possibilities instead of problems, displayed a marked willingness for helping others, and they initiated work, improvements, and arrangements. Not all champions possessed all qualities, but informants reported these characteristics as easily observable in champions, making them very valuable for the organization for promoting positive organizational culture as well as achieving institutional work.

*“I think what characterizes these champions is that they see possibilities instead of limitations. For instance*,* if you arrive at work and you see that one of your colleagues is sick*,* they say; ‘Ok*,* we have to get through together*,*’ ‘Maybe we can do it like this instead’. They take responsibility…. It is these Yes-people”* (Focus group interview, Nursing home, Pre intervention, Site 4).

## Discussion

Explication of the mechanisms that influence organizational functioning is needed to inform resilient performance of individuals, teams, and leaders within healthcare systems [12, 23]. In this exploratory study, we have applied an accepted socio-cognitive framework to existing data related to organization resilience and the role of champions. Resilient performance, as reflected in the introduction, is the capacity to adapt to upcoming and continuous challenges, in order to provide quality care [1]. Resilience is not a static property of an organization but rather reflects dynamic responses, performances and capacities important for adapting to challenges with an end goal of providing quality care. Champions fostered and leveraged connections and relationships among team members, between team members and leaders, as well as between different stakeholders. Confident in their role and organizational support to self-regulate, champions contributed to innovation and led their team to adapt care and roles in altered circumstances to maintain institutional work. In order to do this work, leaders must provide champions “room of manoeuvre” and the necessary autonomy to perform their magic. On the flip side, as champions go the extra mile to cover up for system deficiencies, like lack of resources, champions can end up masking system deficiencies which in the end might result in being a barrier to resilient performance [24]. Furthermore, having to perform continuous adaptations without changing low value conditions might reduce champions motivation for enacting resilience, and instead lead to burnout for the champions [25].

As subject matter experts, champions leveraged their interpersonal skills and abilities to motivate others to higher resilient performance. The personal characteristics of champions included enthusiasm, competence, engagement, willingness to communicate openly, a can-do attitude, and they contributed to positive workplace culture by role-modelling inclusiveness and respect. SDT claims that these personal characteristics only surface when people are in an environment that satisfies their basic psychological needs [16], reaffirming the need to understand how to better support perceived need-satisfaction in the workplace. Implications for leaders in supporting the role of champions for resilient health system performance are discussed in relation to perceived relatedness, autonomy, and competence.

The role of champions as a means of social influence within organisations is well established [4, 5, 26]. In this study within hospitals, nursing homes, and home care services, champions were relied upon to lead aspects of team functioning, uphold leaders’ directives, socialise organizational norms and standards, supervise less competent peers, and foster positive work culture. To perform well in this role, champions built, and drew from, team connectedness. Connectedness is a construct valued in leadership studies, particularly in inclusive and authentic approaches [27], and in resilient health system performance, to cultivate and strengthen good working relationships within the organization [10]. SDT predicts that if warmth and connectedness are not present, exclusion and alienation will occur [16], diminishing individual and team performance and motivation [17]. Our analysis showed that to foster connectedness, leaders and champions needed to be visible and approachable and foster their own relationship with team members, just as team members needed to foster good relationships with one another. Such relationships are key enablers for coordination, involvement, cooperation, and information flow, all of which have been found to promote the resilient performance of a system [3, 28]. A good climate of trust is fundamental in voluntary relationships, built on confidence that individuals will act reliably in concert with moral motives and norms [29]. In this analysis, leaders needed to be seen to be practicing interpersonal justice (perceived respect), and procedural justice (providing valued opportunities to all team members). As one of the central tenets of SDT, the concept of relatedness provides an important key in teamwork and organisational functioning [14, 16]. SDT also predicts creativity and engagement are strengthened when individuals experience perceived autonomy [15].

Leaders can support champions’ perceived autonomy by offering some choice in the activities the champion wishes to engage with, provide opportunity for them to show initiative, and deliver workload flexibility so that champions can flex their activity around the variable clinical demands of their day-to-day work. The importance of local decision-making and self-regulation to support resilient health system performance was demonstrated most recently through the COVID-19 pandemic. A high level of local agency was afforded frontline staff to flexibly evoke radical change [30]. This shift from a centralised top-down model to enable decisions to be taken at a more local level empowers individuals and teams to adapt nimbly when wicked problems arise [9, 31]. As such, even within an overburdened healthcare system during the pandemic, individual healthcare workers were still able to provide appropriate care for their patients in novel ways [23, 32]. SDT predicts such self-regulating locus of control is associated with more intrinsic forms of motivation, implicit in employee wellbeing and performance quality, compared with ill-being, lack of motivation, and poor performance, through external regulation and non-regulation [16, 33–35]. Collaborative studies indicate that ‘middle’ models, that combine a top-down and bottom-up approach might foster the most fruitful outcomes in everyday work [36]. There is also some literature that suggests champions may falter when required to shift their work organization-wide or outside their professional culture [5], reaffirming the important role of leaders in monitoring and supporting champions to experience competence so that they continue to feel effective in what they do.

Leaders in this study were seen as vital contributors to motivate and preserve champions in the organization, and some expressed a desire to know how to foster motivation more broadly in the organization. The role of leaders in fostering self-determined motivation is established in the literature through studies of leadership styles [27] and through studies of motivation styles [13] and application of SDT to leadership more explicitly [37]. It may be unrealistic for leaders to support the same level of psychological need in every instance, or to reach every individual within the organization. Teaching these principles to leaders, champions and followers, may be a means whereby behaviour that fosters motivation and resilient performance more broadly in the organisation can be promoted, and is an area for further research.

### Practical implications

Based on the findings of this study, the following recommendations for leaders and organizations are found valuable to support the motivation of champions in contributing to resilient performance.

- Good working conditions: The presence of good working conditions was found to support champions’ motivation. Leaders, like all employees, must take responsibility for creating a positive work environment where mutual support and cooperation are encouraged.
- Leaders’ encouragement. Leaders’ encouragement is crucial for maintaining champions’ motivation. While this applies to all employees, champions who contribute extra efforts to ensure resilient performance should have their efforts valued and appreciated.
- Facilitation of knowledge sharing: Champions play a key role in facilitating knowledge sharing through training, mentoring, introducing new knowledge and engagement in practice-based learning. To support these efforts, leaders should provide learning arenas where knowledge sharing can occur, such as structured weekly or monthly meetings, informal daily opportunities to share knowledge, and mentoring programs.
- Flexibility and leeway: Champions need flexibility and leeway to perform their extra efforts. This is typically a leader’s responsibility. Given the ongoing restrictions on healthcare resources and increasing demands, it is necessary to develop awareness of how existing meeting arenas are used and to create new ones as needed.
- Valuing Initiatives: It is important to value initiatives from champions. Leaders, managers, and colleagues should consider input and feedback on practice modifications, need for new interventions, and the de-implementation of wasteful practices to improve resilient performance.

### Strengths and limitations

This study was a secondary analysis of existing data undertaken in three different settings. Data collection was not aimed at revealing aspects of team function and leadership or motivation of champions, so there are likely many important individual team and organisational dynamics to be uncovered. Each site may have impacted individuals differently, so there would also be value in undertaking separate analyses in homogenous settings. While we detected differences across sites, this was not mainly due to them coming from different healthcare settings, such as nursing homes or hospitals, but rather the differences laid in the structures, leadership and culture within the different units. Interviews were conducted in Norwegian which may mean some important semantics were lost in translation to English, but care was taken to assure the initial meaning. Perceptions often mapped to more than one SDT construct, so were mapped to all those that were relevant, and thematically categorised within the prevailing construct. Need-satisfaction was predominant in this analysis. It may be that the role of champions and their performance is a predominantly positive phenomenon, but given critical healthcare worker burnout and its association with diminished motivation [33, 38] exploration of need-frustration would be an important addition to the literature. Nevertheless, we have used the perceptions of related to autonomy-, competence- and relatedness-need satisfaction to illuminate supportive practices to promote resilient performance within organizations, and the promise of SDT as an organizing framework. Trustworthiness was ensured through engagement over time in the empirical setting, and observations in workshops [39]. Furthermore, analytical workshops and discussions of the analytical process and coding with all authors included ensured confirmability of the findings [39].

## Conclusion

Self-determination theory helped to provide understanding of autonomous motivation of champions found acting as a capacity for resilient performance in organizations. All three constructs of autonomy, competence, and relatedness were evident in the data. In addition to the SDT themes, an additional theme, called personal characteristics, emerged inductively from the data.

Through the exploration of champions’ autonomous motivation for acting as organizational champions to ensure resilient performance in healthcare organizations, this study provides a more holistic picture where both sides of the story can be understood. Such holistic knowledge of champions is key to understand how to identify, support and maintain champions in their role to lay the groundwork for resilient performance in healthcare.

## Supplementary Information

Supplementary Material 1.

Supplementary Material 2.

Supplementary Material 3.

## Data Availability

The data used for the analysis of this study is available from the corresponding author on reasonable request.
