# Supplementary material for: Two sides of the story: bridging organizational and individual resilience - a qualitative study
Source: BMC Health Serv Res. 2025 Aug 9;25:1050. doi: 10.1186/s12913-025-13013-z (PMC12335771; doi:10.1186/s12913-025-13013-z)
Supplement: Supplementary file 1 — Supplementary Material 1. [file 12913_2025_13013_MOESM1_ESM.docx]

Interview guide BEFORE testing (staff, leaders, authorities)

**Note: Ask for specific examples as a follow-up on all questions where it is appropriate.**

Also: Try to get them talking about WHAT they do and not just what they intend or want to do.

# Introduction:

- Can you talk a little bit about yourself? (age, formal education, current position, and work experience)
- Can you say something about what you think resilience in healthcare is?
- Do you have some examples of how you work to create resilience in your unit/department?

# Work practices:

- Structure
  - Which systems/structures/tools are important for you to be able to do your work well? Please give examples from your own unit.
  - Which local adaptations do you do to ensure that you have access to necessary information and to document in a good way?
- Competence
  - How do you as leaders facilitate competence development for your employees?
  - Do you have examples of what you do to optimize the use of employees' competence and experience?
- Learning
  - How do you promote learning from each other in your unit/department, also across levels and professions?
  - What efforts do you make to learn from what goes well?
  - How do you facilitate possibilities for exchanging and learning new knowledge? (e.g., access to arenas for the exchange of knowledge and experiences, systematic education and training, working across professions and levels, simulation activities)
  - Do you focus on learning from everyday activities (e.g., between student and nurse, through reflection between colleagues, internal knowledge exchange), and if so, how?
- Alignment
  - Can you give examples of what efforts you make to adapt to different situations? (e.g., in the case of new or unexpected events, new requirements/ procedures/ guidelines, local adaptations of external demands)
- Coordination
  - How do you facilitate information flow and collaboration on your ward/unit?
- Risk awareness
  - Can you give examples of what efforts are made to identify and assess risk factors?
  - What do you do prevent risk factors developing into adverse events?
- Leadership
  - As employees, how are you supported and motivated to create a culture where everyone feels included?
  - In what ways are you given the possibility to take on responsibility?
  - How is initiative and new ideas about change and quality improvement received in your department?
- Involvement
  - Can you give examples on how you involve patients and informal carers in your day-to-day work?
  - How are patients and informal carers used as a resource in your day-to-day work? What kind of roles do they have, what do they contribute?
  - What does this type of involvement depend on? Is it the type of task, patient factors, or informal carer factors?
  - How do you work to achieve patient-centered care and positive patient experiences?
- Champions (facilitators)
  - Are there champions in your organization/team that want to take on extra responsibilities and tasks, who get involved in the training of others, or have a special interest in professional matters?
  - How are champions identified in the organization?
  - Do champions get sufficient space to perform and further develop their activities?
- Communication
  - How do you facilitate for communication and exchange of information across professions/departments/units?
  - How do you facilitate for communication and exchange of information with patients and informal carers?
  - Do you have examples of situations where the communication was experienced as particularly successful? What contributed to this success?

# To end:

- Can you talk about your expectations and thoughts on using the new RiH tool in your workplace? How do you envision yourself using the tool? (Who will be involved? Possible challenges, assumed utility, etc.)
- Is there something you want to add about these themes that was not covered by the questions?

# Additional questions for leaders (managers):

- How do you work to support and motivate your staff, and contribute to create a culture where everyone feels included?
- How do you receive and treat suggestions about changes and quality improvement measures from your staff?
- What efforts are made to give staff the opportunity to contribute and take on new responsibilities?
- What efforts are made to create change/learning, not just locally but also at the system/organizational level?
- How can leaders provide an overview over the departments’ champions and resource persons to the other staff?

# Additional questions for the authorities:

- How do you assess organizations according to the ten resilience capacities?
- What efforts do you make to understand adaptations in work practice?
- How do you handle variation in work practices and the need for units/departments to make local adaptations?
- What need do you see for this approach, with its focus on learning from what works well?
- What kind of challenges does this approach entail for your mandate and your engagement with the services out there?
- What do you see this approach being able to contribute to?
