# Supplementary material for: Two sides of the story: bridging organizational and individual resilience - a qualitative study
Source: BMC Health Serv Res. 2025 Aug 9;25:1050. doi: 10.1186/s12913-025-13013-z (PMC12335771; doi:10.1186/s12913-025-13013-z)
Supplement: Supplementary file 2 — Supplementary Material 2. [file 12913_2025_13013_MOESM2_ESM.docx]

Interview guide AFTER testing (staff, managers and authorities)

# Introduction:

- Tell us about yourself (age, formal education, current position and work experience)
- Can you briefly tell us how you have used the RiH tool at your workplace?
- What parts of the tool have you focused on and why? (Any specific or multiple scenarios? Just the scenario function? Just the mapping function?)
- What was your role in the use of the tool?
- Can you say something about what you think resilience in healthcare and adaptiv capacity is all about?

# Learning tool content:

- What are your thoughts on the tool in general? Can you give examples of what you thought worked/didn't work? (technical, content, practical)
- Why have you succeeded/not succeeded in testing the tool?
- What do you think the use of the tool has contributed to?
  - For example: Increased awareness of adaptive capacity/resilience capacities? The importance of context? The importance of involvement?)
- Are there any topics/areas of the tool that you think are particularly relevant or less relevant?
- Do you understand the content of the 10 different resilience capacities? (Structure, leadership, involvement, risk awareness, learning, competence, alignment, communication, coordination, champions (facilitators?))
- Have you made any changes to how you work or think when you're at work? If so, how? (Feel free to give examples based on the different capacities.)
- Is the content of the tool (statements, scenarios) relevant to your everyday work and tasks? Feel free to give examples.
- How do you think the tool can be improved? ((1) acceptability, 2) fit for purpose, and 3) feasibility?)

# Pedagogical approach:

- How did you experience working together in groups? What worked well/what was difficult? (Group dynamics, practical implementation, number of participants.)
- Has the tool contributed to reflection across different groups and levels? If so, how?
- Do you now see other opportunities to reflect and learn from everyday situations? Where and when do you discuss and reflect on the adaptations you make?
- What is needed to increase the learning outcome of the tool’s activities?

# PSI/involvement:

- Have you changed anything regarding how you involve patients and informal carers in your day-to-day work? What activities are they involved in? What role do they have and what do they contribute to in your day-to-day work?
- What role do you feel that patients/informal carers have in adaptation of services and provision of high quality care? Have you changed your opinion regarding the role of patients and informal carers?
- Has anything changed since using the learning tool regarding how you think about and practice regarding the involvement of various internal and external stakeholders (patients, informal carers, interest organisations, other professional groups, etc.)?

# Additional questions for leaders (managers):

- What efforts did you make to get your staff to use the tool?
- How did you arrange for the tool to be used as part of everyday work and how was this maintained over time?
- How have you gone about integrating any changes made at the system/organizational level?
- How did you experience the training in how to use the tool and what improvements can be made, if any?

# Additional questions for the authorities:

- How will this tool and its content be relevant to incorporate into your work?
- How will this change the way you work?
- How do you think this approach can help strengthen your work with the practice field and understand how units and organisations operate?
- How can the benefits of this tool be transferred to health authorities in other countries?
