# Supplementary material for: Two sides of the story: bridging organizational and individual resilience - a qualitative study
Source: BMC Health Serv Res. 2025 Aug 9;25:1050. doi: 10.1186/s12913-025-13013-z (PMC12335771; doi:10.1186/s12913-025-13013-z)
Supplement: Supplementary file 3 — Supplementary Material 3. [file 12913_2025_13013_MOESM3_ESM.docx]

Observation Guide

Testing and Evaluation of the RiH Learning Tool:

# General Information About the Team

• How many participants?

• Which professional groups are represented?

• Where is this taking place? What kind of meeting venue?

# Structure of the Learning Tool

• How do they use the learning tool?

• How do they experience the accessibility/functionality? (navigating, logging in, understanding what to do)

• Are there features they don’t use/can’t manage to use?

• In what way is the learning tool presented (computer screen, projector, printouts, etc.)?

• Which part of the learning tool is being reviewed in this meeting?

• How long does the review take?

# Content of the Learning Tool

• Are they able to understand the content and thus discuss the 10 different resilience capacities?

• How do the various steps in the learning tool work? What seems most useful?

• Is the language understandable to the participants?

• Is the content perceived as relevant? What themes are they working with?

• How do they experience learning from what goes well and from normal work practices?

• How is the patient and family member role discussed in the work with the 10 themes? Is this discussed also outside the theme of involvement? If so, which ones and how?

# Interaction

• Who is responsible for the process/leads the discussion?

• Describe the group dynamics.

• What contributes to the group dynamics functioning/not functioning?

• What role does the researcher play in the workshop? (if the researcher is leading the workshop)

• How well do they manage to create discussion and collaboration across professional groups and levels?

• Which areas of the tool take up the most time? Why?

• What triggers the most engagement among participants?

# Reflection

• How is resilience and the quality perspective understood?

• Does the way quality in healthcare is discussed change through the reflection process?

• Are participants able to relate the areas to situations in their own practice? How?

• Do participants manage to see connections between the different themes? How?

• Is resilience/adaptability described as a useful perspective in healthcare?

• Is there a transfer of solutions/ideas/practices between participants?

• How does the tool contribute to reflection on patients’/stakeholders’ contributions to service adaptation and quality?
